# Pregnant after assisted reproduction: a risk pregnancy is born! 18-years perinatal outcome results from a population-based registry in Flanders, Belgium

**Published:** 2016-12

**Authors:** W Ombelet, G Martens, L Bruckers

**Affiliations:** Genk Institute for Fertility Technology, Department of Obstetrics and Gynaecology, Schiepse Bos 6, 3600 Genk, Belgium; Faculty of Medicine and Life Sciences, Hasselt University, Martelarenlaan 42, Hasselt, Belgium; SPE (Study Center for Perinatal Epidemiology), Brussels, Belgium; Center for Statistics (CENSTAT), Agoralaan building D, 3590 Diepenbeek, Belgium.

**Keywords:** ART, assisted reproduction, ICSI, IUI, IVF, perinatal outcome, pregnancy, pregnancy outcome, singleton, twin

## Abstract

**Background:**

Although the increased risk for perinatal morbidity and mortality of babies born after ART is largely attributed to a higher rate of multiple gestations, a significantly worse perinatal outcome for singleton pregnancies following ART compared to pregnancies after natural conception has been reported as well. Most studies only include IVF/ICSI pregnancies; studies describing the perinatal outcome of pregnancies after non-IVF assisted reproduction are scarce.

**Methods and Materials:**

Population-based cohort study with three exposure groups: a study group of pregnancies (1) after ovarian stimulation (OS), with or without artificial insemination (AI), (2) after IFV or ICSI and (3) a naturally conceived (NC) comparison group. Data from the regional registry of all hospital deliveries in the Dutch-speaking part of Belgium during an 18-years period from January 1993 until December 2010 were used. The perinatal outcome parameters were prematurity, low birth weight, perinatal mortality and morbidity including neonatal intracranial bleeding and need for intubation. Logistic regression analysis was used including mode of conception, female age, foetal sex, parity and year of delivery.

**Results:**

Data on 1 079 814 births were studied: 1 039 415 singletons (19 896 IVF/ICSI, 20 469 OS and 999 050 NC) and 39 041 twins (9 353 IVF/ICSI, 4812 OS and 24 876 NC) were available for analysis. IVF/ICSI singletons had a significantly worse outcome when compared to OS and NC for almost all investigated perinatal parameters. Non-IVF/OS singletons were also significantly disadvantaged for prematurity and low birth weight when compared to NC. The outcome of twin pregnancies was similar for the three groups unless only unlike-sex twins were studied separately. Among this subgroup, IVF/ICSI carried a higher risk for low birth weight when compared to NC. OS unlike-sex twins were at increased risk for low birth weight, intra uterine death and perinatal mortality when compared to NC.

**Conclusion:**

According to our results all ART pregnancies, whether due to IVF/ICSI or non-IVF treatment, have to be considered as risk pregnancies, irrespective of the number of foetuses.

**Limitations of the study:**

Although our logistic regression analysis included co-variables with a potential impact on perinatal outcome such as mode of conception, female age, foetal sex, parity and year of delivery, we couldn't correct for other prominent confounders such as the use of fresh or frozen embryos, use of homologous or donor gametes, smoking, obesity, socio-economic status, occupation exposures and pre-existing disease.

## Introduction

Assisted reproductive technologies (ART) have been increasingly used worldwide since the first infant born over 35 years ago (Steptoe and Edwards, 1978; Adashi et al., 2003; Arslan et al., 2005). In 1993, the number of births after ART in Flanders accounted for 2.4 % of all neonates; 0.9 % were the result of in vitro fertilisation (IVF) whereas 1.5 % were the result of non-IVF assisted reproduction treatment such as ovarian stimulation (OS) with timed intercourse and intrauterine inseminations (IUI) with or without OS (source: Study Center for Perinatal Epidemiology, Brussels). In 2010, 5.7 % of all deliveries in Flanders were the result of assisted reproductive techniques, 3.6 % due to IVF +/- ICSI, and 2.1 % as a result of non-IVF techniques.

Considering this important increase in the use of ART worldwide it will be extremely important to assess the potential health risks for the offspring after ART, not only the perinatal risks but also the longer-term outcomes for children born as a result of ART (Bonduelle et al., 2005, Belva et al., 2007, 2016; Hart and Norman, 2013a, 2013b; Bay et al., 2014).

The major complication of ART is the increased prevalence of multiple pregnancies after IVF/ICSI and non-IVF. Multiple pregnancies are undoubtedly associated with a poorer perinatal and infant outcome (Ombelet et al., 2005; Sazonova et al., 2013; van Heesch et al., 2014). Merritt et al. (2014) reported a 4-5-fold increase in stillbirths among IVF/ICSI and IUI pregnancies when compared to naturally conceived women, mostly due to multiple pregnancies.

Also from an economical point of view multiple- birth infants consume significantly more hospital resources during the neonatal period and the first years of life (Chambers et al., 2007, 2014a, 2014b; Murray and Norman, 2014; van Heesch et al., 2015).

On the other hand, only few differences between outcomes in ART twins compared with twins conceived spontaneously are described (Helmerhorst et al., 2004).

In ART singletons however, an increased risk of birth asphyxia, perinatal mortality, low birth weight and preterm birth was reported (Helmerhorst et al., 2004; Jackson et al., 2004; McGovern et al., 2004; McDonald et al., 2005; Pandey et al., 2012; Stojnic et al., 2013; Ensing et al., 2015; Qin et al., 2016).

The reasons why perinatal health problems occur more frequently in ART- singletons compared to naturally conceived singletons are still unclear and probably diverse (Depp et al., 1996; Dickey et al., 2002; Lambert, 2003; Bonduelle et al., 2005; Pinborg et al., 2005a, 2013; Romundstad et al., 2008).

To avoid multiple pregnancies, reduction in the number of embryos transferred is a reasonable solution (Ombelet et al., 2005; Pinborg, 2005; Boulet et al., 2008; Takeshima et al., 2016; Morimoto, 2016). Elective single embryo transfer (eSET) significantly reduces the risk due to multiple pregnancies compared to double embryo transfer (DET) (Gerris et al., 2002; Thurin et al., 2004; Gerris, 2005; Pandian et al., 2005; Sazonova et al., 2013). Sazonova et al. (2013) also showed that the neonatal and maternal outcome was significantly better for women undergoing two IVF singleton pregnancies compared with one IVF twin pregnancy after DET.

The majority of studies comparing perinatal outcome of ART-babies with naturally conceived newborns are limited to IVF and ICSI methods. For non-IVF ovarian stimulation, registrations of data on perinatal outcome are scarce. Only a limited number of studies reported on the perinatal outcome after intrauterine insemination (IUI) (Nuojua-Huttunen et al., 1999; Wang et al., 2002; Gaudoin et al., 2003; Ombelet et al., 2006; Poon and Lian, 2013; Malchau et al., 2014; Declercq et al., 2015; Stanford et al., 2016).

In the light of the above-mentioned considerations, the objective of the present study was to examine the results of a large cohort of births in Flanders. Therefore we analysed the population-based data of almost all pregnancies and deliveries that occurred in Flanders from 1993 through the end of 2010. We compared perinatal risks between babies born after various infertility treatment procedures (IVF- ICSI and OS) and natural conception. Because an overrepresentation of monozygotic twins can be expected in the naturally conceived comparison group, and since monozygotic twins carry a much higher risk for a poorer perinatal outcome, twin data were reanalysed excluding all same-sex twin pairs.

## Methods and materials

### Study population

In Flanders, the Study Centre for Perinatal Epidemiology (SPE) collects data on the medical and obstetric history, and on perinatal events of each hospital delivery in Flanders of more than 21 weeks of gestational age and ≥ 500 grams at birth. Full voluntary cooperation of all 80 obstetric departments in Flanders has been established since 1993. Flanders represents between 53 and 55 % of all deliveries in Belgium. The average number of births varies between 60 000 and 70 000 deliveries per year. The collected data are based on questionnaires completed by midwifes, obstetricians and paediatricians in the early neonatal period. The obstetric and perinatal file registers 33 items of data per child. All data are sent to a data manager, who carries out a review for errors and omissions. Quality of the data gathering is controlled on a full time basis by checking the incoming records for internal inconsistencies and completeness. Correction and completion is assured by phone calls, additional questionnaires and visits to the local departments. The data manager visits the 80 maternity units ad random to operate a double check. Subsequently the files are stored in a computer database. Annually a complete analysis of the data is published in a national and site-specific report.

### Main outcome measures

The main outcome measures used in this study were gestational age, birth weight, admission to the neonatal intensive care unit (NIC), perinatal mortality and perinatal morbidity including intracranial bleeding and assisted ventilation. Subgroup analyses were made between normal birth weight (> 2500 grams), low birth weight (LBW, < 2500 grams) and very low birth weight (VLBW, < 1500 grams) and between term (≥ 37 weeks), preterm (< 37 weeks) and extreme preterm (<32 weeks) birth. Stillbirth was defined as the birth of a lifeless child of >500g, and neonatal death as the death of a live born child >500g within seven days after birth. Perinatal mortality rate was defined as the sum of stillbirths and neonatal deaths divided by the total number of live and stillbirths. Adequate estimation of the exact malformation rate was not possible since only malformations recognised during hospitalisation were registered.

We also studied the evolution of the multiple birth rates in the study period considering the fact that from July 2003 onwards fewer embryos were being transferred in IVF/ICSI cycles due to a reimbursement strategy implemented by the government (Ombelet et al., 2005). Reimbursement of assisted reproduction technology-related laboratory activities is linked to a transfer policy aiming at substantial multiple pregnancy reduction. This policy was expected to influence the twin delivery rate from May 2004 on.

### Patients

Between January 1, 1993 and December 31, 2010 a total number of 1134864 births could be investigated. The mode of conception was uncertain in 55 070 cases (4.8 %). A total number of 1079814 births were available for analysis. The perinatal outcomes of three different patient groups were compared.

The first group consisted of women who became pregnant after IVF with or without ICSI. Only from 1997 onwards, a distinction was made between IVF and ICSI pregnancies. Consequently all IVF/ICSI singleton and twin births were examined as one group.

The second study group included pregnancies after OS, with or without IUI (intrauterine insemination). In case of artificial insemination, data did not specify factors such as insemination with donor or partner semen, neither for the regimen of controlled ovarian stimulation. The control group consisted of all births following natural conception during the 18-year study period. Perinatal data of singletons, twins and unlike-sex twins were examined independently. Triplet pregnancies accounted for less than 0.03 % of all deliveries and because of their small numbers triplets and higher order gestations were excluded from the study.

### Statistical Analysis

Since a number of factors differ significantly between groups, logistic regression methodology was used to properly correct for these. Logistic regression analysis included co-variables with a potential impact on perinatal outcome such as mode of conception, female age, foetal sex, parity and year of delivery. For the statistical analysis comparing perinatal outcome differences between IVF/ICSI, OS and NC births after adjustment for the different confounding factors, results were presented as odds-ratio (OR), 95 % confidence interval and its corresponding p-values. A difference at the 5 % level of significance was considered the threshold of probability.

## Results

A total number of 1079814 births could be investigated: 1039415 singletons, 39041 twins and 1358 triplets. Table I and II gives an overview of the population characteristics and the perinatal outcome results. For all pregnancies, maternal age was significantly higher (p<0.01) for IVF/ICSI pregnancies compared to OS and NC pregnancies. Logistic regression methodology was used to properly correct for it when comparing ART pregnancy outcome between different groups.

**Table I:** — Population characteristics and perinatal outcome of 1079814 singleton and twin births in Flanders in the period between 1993 and 2010 (NC = Natural Conception, OS = Ovarian Stimulation, IVF = In Vitro Fertilization, ICSI = Intra-Cytoplasmic Sperm Injection).

	SINGLETON (n = 1039415)			TWIN (n = 39041)		
	NC	IVF/ICSI	Non-IVF OS	NC	IVF/ICSI	Non-IVF OS
						
Children births (number)	999050	19896	20469	24876	9353	4812
Maternal age (mean / years)	29.1	32.5	29.9	29.9	32.2	29.8
						
< 32 weeks (%)	0.8	2.0	1.1	9.5	9.6	9.2
>=37 weeks (%)	94.1	89.3	92.4	46.2	43.2	43.6
<1500 gr (%)	0.8	2.0	1.2	9.1	8.8	9.0
>= 2500 gr (%)	95.1	91.3	93.7	45.4	43.2	43.0
Intra-uterine death (%)	0.4	0.8	0.5	1.6	1.1	1.2
Early neonatal death (%)	0.2	0.3	0.2	1.3	1.3	1.7
Perinatal Mortality (%)	0.6	1.1	0.7	2.9	2.4	2.9
Transfer neonatal unit (%)	15.0	20.6	17.9	67.4	69.6	69.1
Endotracheal ventilation (%)	5.1	7.4	5.4	12.1	11.3	11.9
Intracranial bleeding (%)	1.2	2.4	1.4	3.5	3.7	3.2

**Table II:** — Population characteristics and perinatal outcome of 1358 triplet births in Flanders in the period between 1993 and 2010 (NC = Natural Conception, OS = Ovarian Stimulation, IVF = In Vitro Fertilization, ICSI = Intra-Cytoplasmic Sperm Injection).

	TRIPLETS (n = 1358)		
	NC	IVF/ICSI	Non-IVF OS
			
Children births (number)	364	511	483
Maternal age (mean/years)	30.0	30.8	28.9
			
< 32 weeks (%)	35.1	30.3	26.4
>= 37 weeks (%)	3.6	4.1	2.3
<1500 gr (%)	37.9	32.4	27.5
>= 2500gr (%)	3.3	7.6	5.2
Intra-uterine death (%)	4.9	2.0	2.7
Early neonatal death (%)	5.5	1.8	5.7
Perinatal Mortality (%)	10.4	3.7	8.3
Transfer neonatal unit (%)	93.1	95.5	96.4
Endotracheal ventilation (%)	26.2	27.3	24.5
Intracranial bleeding (%)	6.2	4.3	7.9

**Fig. 1:**
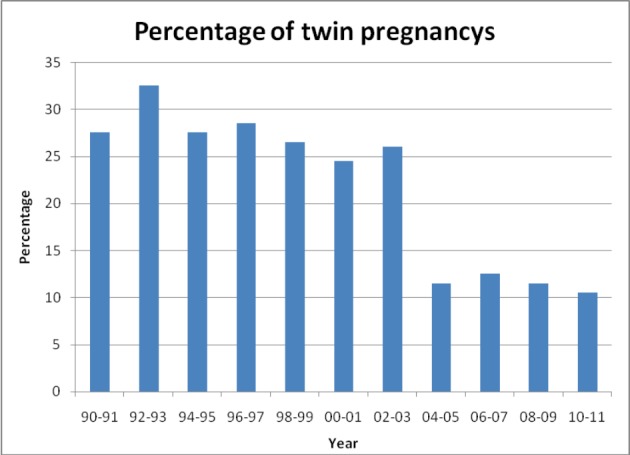
— Effect of Belgian reimbursement policy on the number of twin pregnancies from 1990 until 2011. Significant reduction of twins from 2004 on, the reimbursement policy started on 01-07-2003.

The multiple pregnancy rates for IVF/ICSI dropped from 34.0 % in 1993 to 12.9 % in 2010 (Fig. 1). For OS the multiple pregnancy rates dropped from 14.3 % in 1993 to 8.6 % in 2010. For IVF/ICSI, the proportion of twin and triplet deliveries decreased slowly between 1993 and 2003. In 2004 a sudden decline was noted due to the introduction of the Belgian reimbursement policy. In 2002, 2003 and 2004 the multiple pregnancy rate for IVF/ICSI was 25.4 %, 22.4 % and 11.9 % respectively. The drop between 2003 and 2004 was highly statistically significant (p<0.0001). The OR of the multiple pregnancy rates for the different groups in 2003 versus 2004 was estimated based on a logistic regression model with an unstructured time effect. The OR equalled 2.151 with 95% confidence interval [1.75, 2.65], p < 0.0001.

Triplet and twin pregnancies are at increased risk for perinatal health problems when compared to singletons. The more foetuses involved, the higher perinatal risk (Table I, Table II).

**Fig. 2:**
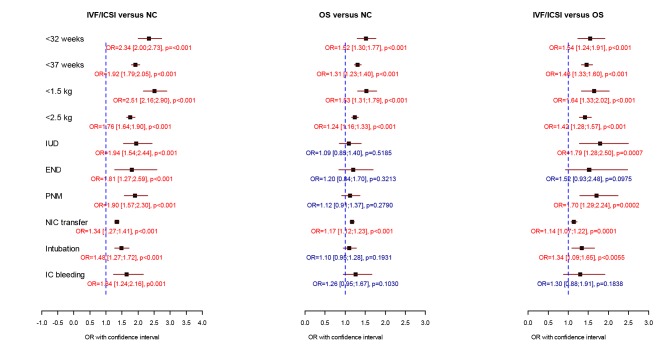
— Comparison of perinatal data of 19869 IVF/ICSI, 20469 non-IVF OS and 999050 spontaneously conceived singleton births. Logistic regression analysis was performed including mode of conception, female age, foetal sex, parity and year of delivery. (OR = odds ratio with 95 % confidence intervals, OS = ovarian stimulation, NC = natural conception, IUD = intrauterine death, END = early neonatal death, PNM = perinatal mortality, NIC transfer = transfer to the neonatal intensive care unit, IC bleeding = intracranial bleeding). * p < 0.05 = significant

**Fig. 3:**
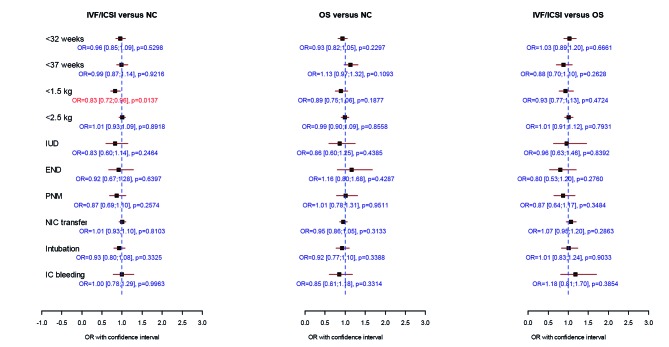
— Comparison of perinatal data of 9353 IVF/ICSI, 4812 non-IVF OS and 24876 spontaneously conceived twin births. Logistic regression analysis was performed including mode of conception, female age, foetal sex, parity and year of delivery. (OR = odds ratio with 95 % confidence intervals, OS = ovarian stimulation, NC = natural conception, IUD = intrauterine death, END = early neonatal death, PNM = perinatal mortality, NIC transfer = transfer to the neonatal intensive care unit, IC bleeding = intracranial bleeding). * p < 0.05 = significant

**Fig. 4:**
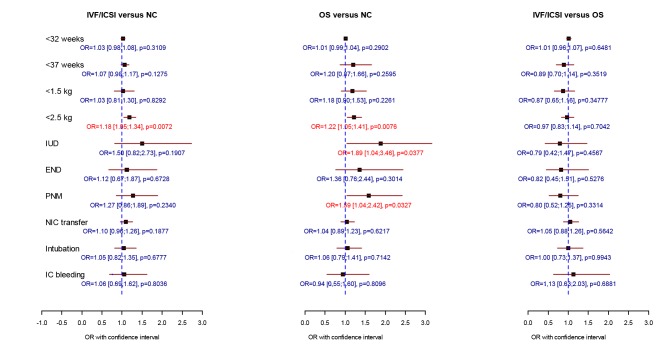
— Comparison of perinatal data of 4203 IVF/ICSI, 2106 non-IVF OS and 7214 spontaneously conceived unlike-sex twin births. Logistic regression analysis was performed including mode of conception, female age, foetal sex, parity and year of delivery. (OR = odds ratio with 95 % confidence intervals, OS = ovarian stimulation, NC = natural conception, IUD = intrauterine death, END = early neonatal death, PNM = perinatal mortality, NIC transfer = transfer to the neonatal intensive care unit, IC bleeding = intracranial bleeding). * p < 0.05 = significant

When compared to IVF/ICSI singletons, IVF/ICSI twins had a 4 to 5-fold increase for extreme prematurity and VLBW and a 2-fold increase in perinatal mortality. IVF/ICSI triplet pregnancy carried a 3 to 4-fold risk for perinatal mortality and a 15-fold increase in extreme prematurity compared to ICF/ICSI singletons. As expected, twins and triplets were responsible for a significantly worse perinatal outcome when compared to natural conception babies in all three investigated study groups (Table I, Table II, Fig. 2, Fig. 3, Fig. 4).

Because of the limited number of triplet pregnancies (n=1358) only the statistical outcome results of singletons and twins are presented.

### Singletons

All perinatal outcome measures were significantly worse when IVF/ICSI births were compared to the NC group. Prematurity, extreme prematurity, low and very low birth weights and a NIC transfer were observed significantly more often after OS compared to NC pregnancies. With the exception of early neonatal death and intracranial bleeding, all investigated parameters were found more often in the IVF/ICSI group when compared to the OS group (Table I, Fig. 2).

### Twins and unlike-sex twins

The perinatal parameters were almost similar for all twins in the different groups with the exception of very low birth weight, which occurred more often in the NC group compared to the IVF/ICSI group (OR = 0.83 [0.72-0.96], p = 0.0137) (Table I, Fig. 3). Since more monozygotic twins are to be expected in the naturally conceived comparison group, data were reanalysed after excluding all same-sex twin pairs. Consequently 7214 unlike-sex naturally conceived twin babies could be compared with 4203 IVF/ICSI and 2106 non-IVF OS twins. When OS unlike-sex twins were compared with NC controls, we observed a significantly higher risk for low birth weight, intra-uterine death and perinatal mortality. Comparing IVF/ICSI with OS births, no differences in perinatal outcome could be shown. Low birth weight was significantly more often seen after IVF/ICSI versus NC (Fig. 4).

## Discussion

A major complication of assisted reproduction is a multifoetal pregnancy (Callahan et al., 1994; Bergh et al., 1999; Ombelet et al., 2005; Sazonova et al., 2013; Merritt et al., 2014; van Heesch et al., 2014). Not only neonatal complications and associated long-term sequelae, but also maternal complications are seen more often after multiple births (Collins, 2002; Ombelet et al., 2005).

The data presented in Table I and II confirm the well-known negative effect of multiple pregnancies on perinatal outcome.

US and European IVF/ICSI results are published on an annual basis. In 2010 the multiple pregnancy rate after IVF/ICSI was 20.6 % in Europe (19.6 % twins and 1.0 % triplets) while in the United States 43.4% of ART-conceived infants were twin infants, and a smaller proportion (3.0%) were triplets and higher order infants (Sunderam et al., 2013; Kupka et al., 2014). According to the Latin American Registry the prevalence of twins and triplets was still as high as 20.7 % and 1.09 % in fresh autologous IVF/ICSI cycles in 2013, a very alarming figure (Zegers- Hochschild et al., 2016). Beside Scandinavian countries, Japan is one of the exceptional countries where even without a stringent reimbursement policy multiple pregnancy rates dropped significantly, only because the IVF centres realised to understand the message that the aim of ART is to obtain healthy babies and not to achieve the best success rates (Morimoto, 2016). In 2012 single-embryo transfer accounted for 82.6 % of all transfers performed in Japan (Takeshima et al., 2016).

In Flanders, the multiple pregnancy rates for IVF/ICSI and non-IVF OS declined significantly during the study period (Fig.1). A significant drop of multiples was observed in 2004 reflecting the impact of a new embryo transfer strategy that was implemented by the health authorities in July 2003 (Ombelet et al., 2005). In 2003 all licensed IVF centres agreed to a government proposal in which reimbursement of IVF/ICSI laboratory costs would be linked to a reduction of embryos transferred after ART. National Belgian registration data show that reimbursement of IVF laboratory costs coupled to a legal limitation in the number of embryos transferred, were associated with a more than 50% reduction of the multiple pregnancy rate from 27 to 11% without reduction of the pregnancy rate per cycle, and with an increase in the number of fresh and frozen ART cycles due to improved access to treatment (de Neubourg et al., 2013).

For non-IVF ovarian stimulation, registration of data on perinatal outcome is scarce. In a Finnish study a similar perinatal outcome was observed for IUI compared to spontaneous and IVF pregnancies (Nuojua-Huttunen et al., 1999). In a second study singleton IUI births were about 1.5 times more likely to be born preterm than NC singletons, whereas the IVF/ICSI group was 2.4 times more likely to be born preterm than the NC group (Wang et al., 2002). Gaudoin et al. (2003) using the Scottish national data reported a poorer perinatal outcome of singletons after OS but only when IUI was done with partner’s semen. We previously reported the results of births following non-IVF hormonal treatment using the 1993-2003 SPE data (Ombelet et al., 2006). For OS-singletons a significantly higher incidence of prematurity, LBW, VLBW and most neonatal morbidity parameters was observed. Twin pregnancies resulting from OS showed an increased rate of neonatal mortality, assisted ventilation and respiratory distress syndrome. After excluding same-sex twin sets, OS twin pregnancies were at increased risk for extreme prematurity and VLBW.

In the present study the perinatal results of a large cohort of IVF/ICSI, OS and NC births were examined, making use of the SPE data. Our results showed that IVF/ICSI singletons had a significantly worse outcome when compared to OS and NC for almost all investigated perinatal parameters. Non-IVF OS singletons were also significantly disadvantaged for prematurity and low birth weight when compared to NC. This is contradictory to the results obtained in a large Japanese retrospective cohort study showing that the perinatal risks found among singletons born after ART were similar whatever type of ART was used (Hayashi et al., 2012).

To highlight the excellent quality of our SPE- registration in Flanders we can put forward the following arguments: the cohort is drawn prospectively, all maternity units in Flanders are participating since 1993, a very low loss to follow-up rate, the accuracy of the gathered data which are checked on a regular basis, the data manager (GM) responsible for the data remained the same for the whole study period and adjustment for the most important factors influencing perinatal outcome is easily possible due to the large cohort of data. On the other hand, the weaknesses of the study were the following: correcting for other prominent confounders such as smoking, socio-economic status, occupation exposures, pre-existing disease, etc. was not possible. Neither did the SPE-data allow us to differentiate between inseminations with donor or partner sperm. On the other hand, pregnancies resulting from donor insemination carry no increased perinatal risk compared to spontaneous gestations (Nuojua-Huttunen et al., 1999; Hoy et al., 1999; Wang et al., 2002; McDonald et al., 2005). Therefore we postulate that the poor perinatal outcome observed for non-IVF OS births would have been worse if only pregnancies following IUI with partner’s semen were included.

In the IVF/ICSI group it was not possible to differentiate pregnancies with fresh or frozen embryos. This can influence the outcome results, especially when talking about birth weight. Different studies have proven that macrosomia (birth weight > 4500 grams) and LGA (large for gestational age defined as birth weight > 2 standard deviations above the reference value for gestational age and sex) are significantly increased for infants born after FET (frozen embryo transfer) compared with fresh embryo transfer cycles (Maheshwari et al., 2012; Wennerholm and Henningsen, 2013; Hansen and Bower, 2014; Pinborg et al., 2014). According to the Belgian Registry 32 % of all deliveries after IVF/ ICSI were the results of transferring frozen/thawed embryos in 2010 (BELRAP, 2012). Belgian data indicate that for all IVF/ICSI babies born during the study period, between 20 and 30 % were the result of transferring frozen/thawed embryos. Data from a Danish National cohort study (2007-2012) showed that singletons born after IUI had an increased risk of adverse perinatal outcomes compared with NC children, similar to ICSI, but favourable outcomes compared with IVF. Stimulation with clomiphene citrate was associated with higher risk of LBW compared with natural-cycle IUI, but follicle- stimulating hormone treatment did not seem to be associated with adverse outcomes. (Malchau et al., 2014). The type of ovarian stimulation was not known in our study cohort.

It remains to be explained why the perinatal outcome of ART singletons is worse compared to NC singletons, as shown in this study. According to a systematic review and meta-analysis of Pinborg et al. (2013) subfertility is a major risk factor for a poor perinatal outcome in ART singletons, even when compared to naturally conceived siblings of the same mother. This means that factors related to the ovarian stimulation and/or ART methods per se also play a major role. Other causes such as parental characteristics and higher maternal age, the in-vitro techniques, culture media, and possibly additional freezing or vitrification procedures may play a role as well (Henningsen and Pinborg, 2014).

Above this, ART singletons are also more frequently the result of spontaneous foetal demise (vanishing twins) or selective foetal reduction after infertility treatment. In both circumstances a higher risk for adverse obstetric and perinatal outcome compared to naturally conceived singletons has been described (Depp et al., 1996; Dickey et al., 2002; Schieve et al., 2002; Lambert, 2003; Pinborg et al., 2005b; Sazonova et al., 2011). On the other hand, it has been shown in a population-based cohort study in Norway that adverse outcome such as birth weight, gestational age, risks of small for gestational age babies and preterm delivery did not differ among infants of women who had conceived both spontaneously and after assisted fertilisation. The adverse outcomes after ART could therefore be attributable to the factors leading to infertility itself, rather than to factors related to the reproductive technology (Romundstad et al., 2008). In a population-based study of 272551 women, Seggers et al. (2016) studied the birth weight in consecutively born sibling singletons conceived with and without in vitro fertilization (IVF) to disentangle the effects of maternal characteristics from those of the IVF treatment itself. They concluded that maternal characteristics of subfertile women are associated with a lower birth weight. IVF treatment itself did not additionally contribute to a lower birth weight in the offspring (Seggers et al., 2016).

For twins, differences in perinatal outcome between IVF/ICSI, OS and NC pregnancies, as observed in singletons, almost disappeared with the exception of extreme prematurity which was observed more often in NC compared to IVF/ ICSI pregnancies (Fig. 3). The higher incidence of high-risk monochorionic pregnancies after NC compared to ART pregnancies is well known (Chow et al., 2001). Therefore we reanalysed our data after excluding all same-sex twin pairs. In this subgroup of dizygotic pregnancies we observed a significant higher risk for low birth weight, stillbirth and perinatal mortality for OS unlike-sex twins compared with NC controls. This was not seen in the IVF/ICSI unlike-sex twins. One of the possible explanations might be that in the OS twin group a higher proportion of high-order multiple pregnancies occur that have subsequently undergone selective foetal reduction. Multifoetal pregnancy reduction (MPR) is associated with a higher risk for preterm delivery and low birth weight (Haas et al., 2016). A retrospective analysis performed on Flanders data showed that OS twins were more likely to be the result of MPR when compared to IVF/ICSI twins (Ombelet et al., 2007). Moini et al. (2012) reported a higher rate of extreme preterm birth, admission to the NIC Unit and perinatal mortality in the unlike-sex twins for IVF/ICSI versus NC. In our study and among this subgroup, IVF/ICSI only carried a higher risk for low birth weight when compared to NC.

Recent reports indicate that the perinatal outcome of children born after ART improve over time (Henningsen and Pinborg, 2014; Henningsen et al., 2015). This can probably be explained by different factors: less multiples because of the increased use of elective single-embryo transfer, better access to IVF-related procedures consequently leading to a shorter duration of infertility before ART is started. A refinement of both clinical and laboratory skills during the past three decades may be another explanation (Henningsen and Pinborg, 2014). In our study we couldn't confirm this improvement of perinatal outcome over time.

## Conclusion

Our results indicate that all ART-pregnancies, whether due to IVF/ICSI or non-IVF assisted reproduction, have to be considered as risk pregnancies, irrespective of the number of foetuses. IVF/ICSI pregnancies undoubtedly have the worse perinatal outcome in singleton pregnancies, not only when compared to NC pregnancies but also when compared to non-IVF ART pregnancies. For twins these differences in perinatal outcome between the three study-groups almost disappeared, for unlike- sex twins the perinatal outcome was less favourable for OS when compared to NC. Multiple pregnancies as such carried a significantly higher risk for complications and worse perinatal outcome when compared to singletons in all study groups.
